# Supplementary material for: Affinity and Specificity for Binding to Glycosaminoglycans Can Be Tuned by Adapting Peptide Length and Sequence
Source: Int J Mol Sci. 2021 Dec 31;23(1):447. doi: 10.3390/ijms23010447 (PMC8745253; doi:10.3390/ijms23010447)
Supplement: Supplementary file 1 [file ijms-23-00447-s001.zip › ijms-1526192-supplementary.pdf]

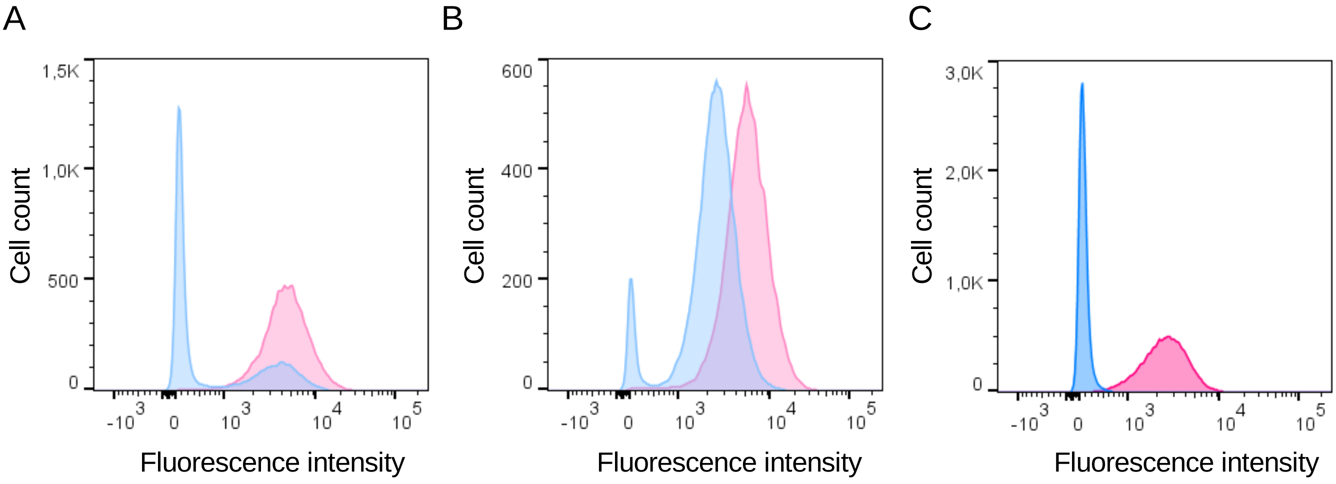


**Figure S1.** Subcloning of pgsB-618 cells results in HS$^+$ (pgsB-618 clone 19) and HS$^-$ (pgsB-618 clone 16) cells. The expression of HS on pgsB-618 (light blue), CHO-K1 (light pink), pgsB-618 clone 16 (blue) and pgsB-618 clone 19 (pink) cells was measured using flow cytometry with a primary anti-HS antibody and a secondary PE-labeled antibody. Histograms are shown with the cell count on the y-axis and the fluorescence intensity of the secondary antibody on the x-axis. The expression of HS was measured at different time points: (**A**) before subcloning, on fresh cell cultures; (**B**) before subcloning, two weeks later; (**C**) right after subcloning.

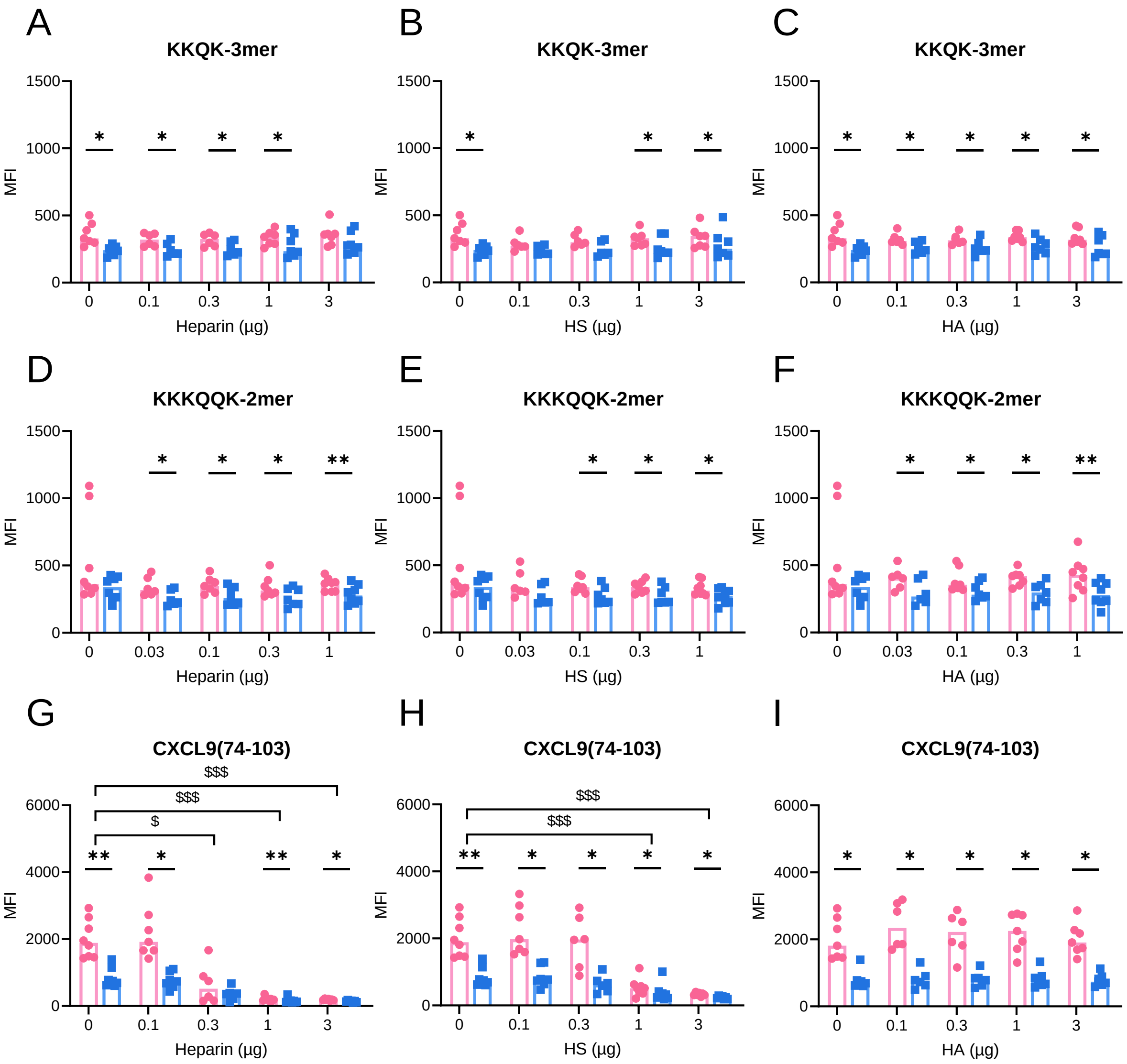


**Figure S2.** Binding of CXCL9(74-103) to cellular GAGs on CHO cells can be significantly decreased by adding heparin or HS. TAMRA-labeled peptides (0.24 nmol; KKQK-3mer (A,B,C); KKKQQK-2mer (D,E,F) or CXCL9(74-103) (G,H,I)) and GAGs (heparin (A,D,G); HS (B,E,H) or HA (C,F,I)) were added to either $HS^+$ (pink) or $HS^-$ (blue) CHO cells, and the binding of the peptide to cellular GAGs was assessed using flow cytometry. Median fluorescence intensity (MFI) is depicted on the y-axis and the amount of added GAG on the x-axis. MFI values were normalized first by subtracting the MFI of the unstained sample (background). (n ≥ 6; Wilcoxon test, * $p < 0.05$, ** $p < 0.01$ (comparison of $HS^+$ and $HS^-$ CHO cells; per peptide and per dose of GAG); Mann–Whitney test, $^{\$}p < 0.05$, $^{\$\$\$}p < 0.001$ (comparison of MFI ($[HS^+]–[HS^-]$) in baseline condition (without GAG) with different doses of GAG; per peptide and per GAG)).
